# Mediators of measurement-based care implementation in community mental health settings: results from a mixed-methods evaluation

**DOI:** 10.1186/s13012-022-01244-1

**Published:** 2022-10-21

**Authors:** Cara C. Lewis, Meredith R. Boyd, C. Nathan Marti, Karen Albright

**Affiliations:** 1 Kaiser Permanente Washington Health Research Institute, 1730 Minor Avenue, Suite 1600, WA 98101 Seattle, USA; 2 Department of Psychiatry and Behavioral Sciences, School of Medicine, & Department of Health Systems and Population Health, School of Public Health, University of Washington, 325 Ninth Street, Seattle, WA 98104 USA; 3 Department of Psychology, University of California Los Angeles, 502 Portola Plaza, Los Angeles, CA 90095 USA; 4 Abacist Analytics, PO Box 11581, Austin, TX 78711 USA; 5 Division of General Internal Medicine, University of Colorado School of Medicine, 1635 Aurora Court, Aurora, CO 80045 USA; 6 Denver-Seattle Center of Innovation, Department of Veterans Affairs, 1700 N. Wheeling ST, CO P1-15180045 Aurora, USA

**Keywords:** Measurement-based care, Depression, Community mental health, Implementation, Sustainment, Mediator, Tailored, Standardized

## Abstract

**Background:**

Tailored implementation approaches are touted as superior to standardized ones with the reasoning that tailored approaches afford opportunities to select strategies to resolve determinants of the local context. However, results from implementation trials on this topic are equivocal. Therefore, it is important to explore relevant contextual factors that function as determinants to evaluate if they are improved by tailoring and subsequently associated with changes in implementation outcomes (i.e., via statistical mediation) to better understand how tailoring achieves (or does not achieve) its effects. The present study examined the association between a tailored and standardized implementation approach, contextual factors that might mediate change, and a target implementation outcome in an initiative to implement measurement-based care (specifically the clinical integration of the Patient Health Questionnaire [PHQ-9] for depression) in a community mental health organization.

**Methods:**

Using a cluster randomized control design, twelve community-based mental health clinics were assigned to a tailored or standardized implementation group. Clinicians completed a self-report battery assessing contextual factors that served as candidate mediators informed by the Framework for Dissemination at three time points: baseline, 5 months after active implementation support, and 10 months after sustainment monitoring. A subset of clinicians also participated in focus groups at 5 months. The routine use of the PHQ-9 (implementation outcome) was monitored during the 10-month sustainment period. Multi-level mediation analyses assessed the association between the implementation group and contextual factors and the association between contextual factors and PHQ-9 completion. Quantitative results were then elaborated by analyzing qualitative data from exemplar sites.

**Results:**

Although tailored clinics outperformed standard clinics in terms of PHQ-9 completion at the end of active implementation, these group differences disappeared post sustainment monitoring. Perhaps related to this, no significant mediators emerged from our quantitative analyses. Exploratory qualitative analyses of focus group content emphasized the importance of support from colleagues, supervisors, and leadership when implementing clinical innovations in practice.

**Conclusions:**

Although rates of PHQ-9 completion improved across the study, their sustained levels were roughly equivalent across groups and low overall. No mediators were established using quantitative methods; however, several partial quantitative pathways, as well as themes from the qualitative data, reveal fruitful areas for future research.

**Trial registration:**

Standardized versus tailored implementation of measurement-based care for depression. ClinicalTrials.gov NCT02266134, first posted on October 16, 2014

Contributions to the literature

- The tailored implementation group demonstrated greater PHQ-9 completion (implementation outcome) than the standard implementation group at the end of the active implementation period, yet this difference disappeared by the end of the sustainment period.
- No significant contextual mediators emerged through rigorous quantitative methods and analyses; only incomplete pathways were observed.
- Exploratory qualitative analyses revealed the critical importance of support from colleagues, supervisors, and leadership when implementing new practices.

## Background

Tailored implementation approaches are touted as superior to standardized ones [1]. The rationale is that “one-size-fits-all,” or standardized approaches, ignore empirical evidence that “context matters” for effective implementation,^1^ whereas a tailored approach affords the opportunity to select and match (i.e., tailor) strategies to the determinants^2^ that serve as barriers to implementation within the local context [1]. A meta-analysis demonstrated superior implementation outcomes for tailored over standardized approaches [4], but more recent results from rigorous implementation trials are equivocal, with some offering null findings, such as the multinational-tailored implementation for chronic diseases (TICD) [5]. In his reflection on TICD, Wensing [5] suggests that perceived determinants and tailored strategies were valid yet insufficient and that emergent determinants may demand adaptation and ongoing tailoring. A related critical unanswered question is whether tailored approaches, that are responsive to relevant contextual factors and resolve those functioning as determinants, improve the likelihood of *sustaining* an evidence-based intervention past an active implementation period.

Tailored implementation can be defined as the process of matching strategies to address determinants (i.e., barriers) within the local context [6]. The idea is that effective tailored implementation occurs by improving or resolving determinants in a given context. This temporal relationship in which the resolution of determinants leads to improvements in intervention implementation can be explicitly evaluated using mediation analyses. Moreover, longitudinal studies in which experimental exposure differentially yields a change in determinants and outcomes can offer confidence in a causal relationship. To this end, relevant contextual factors need to be examined over time to determine if they are improved by tailoring and subsequently associated with changes in target implementation outcomes. 

### Current study

We set out to address pressing implementation questions in an initiative to bring measurement-based care (MBC) into community mental health settings. MBC is the systematic assessment of patient symptoms prior to clinical encounters to collaboratively inform care [7]. MBC can be conceptualized as having three components of fidelity. The foundational MBC component, per its evidence base, requires patient-reported outcome completion, using measures like the Patient Health Questionnaire (PHQ-9), prior to every single encounter [8, 9]. This component alone—often referred to as “routine outcome monitoring” or “progress monitoring”—has been demonstrated to improve clinical outcomes [10, 11], and yet it is met with numerous implementation challenges in community mental health settings where fewer than 20% of clinicians engage in MBC [7]. MBC fidelity is optimized when score trajectories are discussed with patients (second component) and treatment is adjusted according to the data (third component). In collaboration with Centerstone—one of the US largest not-for-profit community behavioral health providers—we designed a study to compare the effectiveness of standardized versus tailored approaches to implementation (details about the activities in each group are found in the “Method” section) to support MBC integration.

Our active implementation phase outcomes of this cluster randomized trial are published elsewhere [12]. We found that tailored implementation outperformed the standardized approach in terms of PHQ-9 measure completion (foundational fidelity component) but overall fidelity remained suboptimal in that score review, and collaborative discussion did not significantly increase in either group and MBC fidelity was not associated with improvements in patient depressive severity outcomes.

### Aims and hypotheses

In the current study, we explored patient-reported PHQ-9 completion beyond this initial implementation phase and examined the degree to which contextual factors served as determinants of this implementation outcome over time. To do so, we used a mixed-methodological approach in which we first conducted a quantitative mediation^3^ analysis, then analyzed qualitative data from four exemplar sites to explore possible novel determinants, and then integrated the two datasets by merging them for analysis and comparison [14]. We hypothesized that tailored implementation would remain superior and that contextual factors (e.g., structure, norms) would emerge as mediators of patient-reported PHQ-9 completion in the tailored group, but not in the standardized group. This evaluation was largely exploratory given the dearth of implementation theory to inform hypotheses about which specific contextual factors might causally lead to the institutionalization of MBC.

## Methods

### Measurement-based care: intervention and outcome

MBC has been shown to improve treatment outcomes compared with treatment as usual and may be particularly beneficial for identifying and adjusting treatment when a patient has experienced a lack of improvement or deterioration [9]. In the current study, the PHQ-9 was used as the measure for routine administration to adult patients with a depression diagnosis [15, 16]. The PHQ-9 is a nine-item self-report measure that assesses depressive symptoms that align with the DSM-5 diagnostic criteria for a major depressive episode; scores reflect the severity of depressive symptoms. The PHQ-9 is a widely used measure with strong evidence of reliability and validity, including sensitivity to change [15, 17]. The extant literature indicates that routine administration of the PHQ-9 alone can lead to benefits over treatment as usual as it reveals patient deterioration or lack of progress [9, 15]. Results from our active implementation phase of this trial indicated that the tailored approach led to improvements in PHQ-9 completion, over that of the standardized approach. Accordingly, PHQ-9 completion was the primary outcome of the sustainment phase of this trial.

### Design

This mixed-methods study was conducted in 12 community-based mental health clinics in two Midwestern states [18]. Participating clinics were assigned to the implementation group using a dynamic cluster randomized design similar to Chamberlain et al. [19]. Clinics were matched on urbanicity, number of clinicians, and number of patients seeking care for depression. Participants were not blind to the study group. All eligible clinicians were offered a 4-h MBC training followed by 5 months of active implementation support. The type of active implementation support was determined by the group. Details of the study protocol and methods have been described previously [20, 21]. This study was reviewed and approved by Indiana University’s IRB; clinician participants provided informed consent.

### Implementation groups

In the tailored group, implementation teams of 5–8 members representing different clinic roles (i.e., clinician, office professionals, directors) were formed and met approximately every 3 weeks for 5 months of active support. Individuals who were identified as influential in the organization using a sociometric network analysis [22] and/or as having positive attitudes toward MBC via a survey [23] or self-nomination were invited to join. Meetings focused on reviewing data on clinic MBC use and strategies to further MBC implementation. Implementation teams could tailor the MBC guideline or subscribe to the standardized recommendation. Several teams opted to expand their guidelines to include individuals 12 years and older given that the PHQ-9 is validated for use with youth. Several teams opted to expand their guideline to include all diagnoses given high rates of comorbid depression in community-based populations. Five of six teams opted to tailor their guideline with details offered in Table 1.

**Table 1:** PHQ9 administration guidelines specified by clinics in the tailored group

Clinic #	State	Guideline tailored?	Target frequency	Age range specified in guideline	Diagnoses specified in guideline
1	TN	Yes	Every session*	21–65	Depression diagnoses
5	TN	Yes	Every session	12 and older	Depression diagnoses
6	IN	No	Every session	21–65	Depression diagnoses
9	IN	Yes	Every session	12 and older	Any diagnosis
10	IN	Yes	Every session	12 and older	Any diagnosis
12	IN	Yes	First week of the month	12 and older	Depression diagnoses

***Note.*** *This site opted to focus on the administration of the PHQ-9 to 10 patients per clinician caseload initially to aid clinicians in gaining experience with measurement-based care before expanding efforts to all patients with a depression diagnosis

In the standardized group, consultation teams were formed and open to all participating clinicians. However, the schedules of individuals who were identified as influential and/or as having positive attitudes were prioritized. Meetings were led by an external consultant and focused on supporting clinician use of MBC as recommended by the study guideline.

### Participants

Clinicians were included if they completed the baseline and 5-month assessment of the contextual measures and had one or more appointments with an eligible patient during the sustainment phase of the study. Of the 155 clinicians with sustainment phase data, 122 had sufficient data on one or more of the candidate measures to be included in the study. See Table 2 for the demographic characteristics of clinicians.

**Table 2:** Clinician demographics (*N* = 122)

		Standardized % (*n*)	Tailored % (*n*)	Total % (*n*)
Gender	Woman	75.0% (42)	87.9% (58)	82.0% (100)
	Man	25.0% (14)	12.1% (8)	18.0% (22)
Race	White	76.4% (42)	95.5% (63)	86.8% (105)
	African American	16.4% (9)	4.5% (3)	9.9% (12)
	Other	7.3% (4)	0.0% (0)	3.3% (4)
Ethnicity	Hispanic/Latinx	3.6% (2)	0.0% (0)	1.7% (2)
	Non-Hispanic/Latinx	96.4% (53)	100.0% (66)	98.3% (119)
Licensure	Currently licensed	51.8% (29)	69.7% (46)	61.5% (75)
	Not licensed	48.2% (27)	30.3% (20)	38.5% (47)
Theoretical orientation	Other	34.5% (19)	43.1% (28)	39.2% (47)
	Cognitive behavioral	65.5% (36)	56.9% (37)	60.8% (73)
Primary Population	Adults	58.9% (33)	65.2% (43)	62.3% (76)
	Youth	41.1% (23)	34.8% (23)	37.7% (46)
		*M* (*SD*)	*M* (*SD*)	*M* (*SD*)
Age		43.73 (12.80)	41.11 (11.10)	42.31 (11.93)

Patient data pertaining to MBC fidelity were included in the sustainment phase if patients met clinic-specific eligibility criteria in the tailored group, or if in the standardized group, they were diagnosed with depression and were at least 21 years old at the time of their session. Data from a total of 6709 patients were included. These patients accounted for 33,354 sessions that served as the target for our primary implementation outcome (i.e., PHQ-9 completion).

### Framework for dissemination

There are numerous determinant frameworks that can guide implementation and evaluation. Across all, it is clear that contextual factors ought to be considered at varying levels of the organization from the beliefs of individuals involved to the broader socio-political conditions in which the organization is embedded. The Framework for Dissemination was used as a guiding framework for the identification and evaluation of a relatively parsimonious set of contextual factors as candidate mediators because it grew from an academic-community partnership, offered a three-phased evaluation process, and integrated the best available evidence [24]. This framework outlines six contextual domains theorized to be associated with successful implementation of a new practice: (i) norms and attitudes: knowledge, beliefs, and values of stakeholders about the intervention as well as perceptions of organizational support for the intervention (i.e., organizational climate); (ii) structure and process: organizational operations and attributes such as decision making processes, types of services, and organization size; (iii) resources: financial, technological, human, social, and political capital that supports implementation; (iv) policies and incentives: the costs and benefits of use of the new intervention as determined by regulatory policies and funding mechanisms; (v) networks and linkages: connections between organizations and between individuals that facilitate knowledge sharing and social support related to the new intervention; and (vi) media and change agents: internal and external sources of credible information and influence such as external trainers, consultants, and internal champions of the intervention.

### Measures

The battery of measures used to capture candidate mediators was comprised of six, self-report instruments completed by clinicians at baseline, after 5 months of active implementation, and again 10 months later (i.e., end of sustainment monitoring). See Table 3 for a description of the measure, sample items for each subscale, and how subscales were mapped onto the Framework for Dissemination. Scale reliability estimates in the current sample are provided in Table 4.

**Table 3:** Measures used to assess candidate mediators

Measure name	Description	Subscale	Sample item(s)	Framework for Dissemination Domain
Attitudes Towards Standardized Assessment (ASA)	24-Item measure of clinician attitudes towards the use of standardized assessment	Clinical utility	Standardized progress measures provide more useful information than other assessments like informal interviews or observations	Norms and attitudes
		Benefit for treatment planning	Standardized progress measures help gather objective information about whether treatment is working	Norms and attitudes
		Practicality	Standardized progress measures can efficiently gather information	Norms and attitudes
Monitoring and Feedback Attitudes Scale (MFA)	20-Item measure of clinician attitudes towards routine progress monitoring and provision of feedback to patients about treatment progress	Benefit	Monitoring treatment progress is an important part of treatment	Norms and attitudes
		Harm	Providing feedback to clients about treatment progress (or lack thereof) would potentially harm the therapeutic alliance	Norms and attitudes
Evidence-Based Practice Attitudes Scale (EBPAS)	15-Item measure of clinician attitudes towards adoption of evidence-based practices; lower scores indicative of worse attitudes	N/A total score used	N/A	Norms and attitudes
Survey of Organizational Functioning (SOF)	162-Item measure divided into seven scales: motivation for change, resources, staff attributes, organizational climate, job attitudes, workplace practices, and training exposure and utilization	Program needs	Your program needs additional guidance in increasing program participation by clients	Resources
		Training needs	You need more training for assessing client problems and needs	Resources
		Pressures for change	Current pressures to make program changes come from program supervisors or managers	Polices and incentives
		Offices	Your offices and equipment are adequate	Resources
		Staffing	There are enough counselors here to meet current client needs	Resources
		Training	This program holds regular in-service training	Resources
		Computer access	Computer problems are usually repaired promptly at this program	Resources
		E-communications	You have easy access for using the internet at work	Resources
		Growth	This program encourages and supports professional growth	Norms and attitudes
		Efficacy	You have the skills needed to conduct effective individual counseling	Norms and attitudes
		Influence	Staff generally regard you as a valuable source of information	Norms and attitudes
		Adaptability	You are willing to try new ideas even if some staff members are reluctant	Norms and attitudes
		Mission	Your duties are clearly related to the goals of this program	Norms and attitudes (climate)
		Cohesion	Staff here all get along very well	Norms and attitudes (climate)
		Autonomy	Counselors here are given broad authority in treating their own clients	Norms and attitudes (climate)
		Communication	Program staff are always kept well informed	Norms and attitudes (climate)
		Stress	Staff frustration is common here	Norms and attitudes (climate)
		Change	The general attitude here is to use new and changing technology	Norms and attitudes (climate)
		Burnout	You feel overwhelmed by paperwork	Norms and attitudes
		Satisfaction	You feel appreciated for the job you do	Norms and attitudes
		Director leadership	My program director takes time to listen carefully to and discuss people’s concerns	Network and linkages
		Peer collaboration	Counselors at this program make a conscious effort to coordinate with other service professionals	Network and linkages
		Deprivatized practice	In the past year, you have received meaningful feedback on your performance from colleagues	Network and linkages
		Collective responsibility	Many counselors in this program feel responsible to help each other do their best	Network and linkages
		Focus on outcomes	When making important decisions, the program always focuses on what’s best for client improvement	Norms and attitudes
		Reflective dialogue	In the past year, you have had frequent conversations with colleagues about what helps clients improve	Network and linkages
		Counselor socialization	Experienced counselors invite new counselors into their sessions to observe, give feedback, etc.	Network and linkages
		Training satisfaction	You were satisfied with the training offered at workshops available to you last year	Media and change agents
		Training exposure	In the last year, how often did you attend training workshops held within 50 miles of your agency?	Media and change agents
		Training utilization-individual level	When you attend workshops, how often do you try out the new interventions or techniques learned?	Media and change agents
		Training utilization–program level	How often do new interventions or techniques that the staff from your program learn at workshops get adopted for general use?	Media and change agents
Implementation Climate Scale (ICS)	18-Item measure of the extent to which employees perceive their organization as prioritizing and valuing the implementation of EBPs	N/A total score used	N/A	Norms and attitudes (climate)
Barriers and Facilitators Scale (B&F)	19-Item measure comprised of three subscales that assess common determinants of implementation. For this study, the referent was edited to be “MBC.”	Job-related structures	Our progress notes support MBC	Structure and process
		Program-level structures	My agency has a committee who oversees how MBC is being done	Structures and process
		Agency leadership support	My agency has a person who is a strong advocate for MBC	Media and change agents
Implementation Leadership Scale (ILS)	12-Item measure comprised of four subscales that assess aspects of implementation leadership	N/A total score used	N/A	Media and change agents

**Table 4:** Means and standard deviations of candidate mediators

Determinant	Standardized			Tailored			
	Baseline	5 Mo	15 Mo	Baseline	5 Mo	15 Mo	Reliability
	*M* (*SD*)	*M* (*SD*)	*M* (*SD*)	*M* (*SD*)	*M* (*SD*)	*M* (*SD*)	*α*
**Norms & attitudes**							
ASA-clinical utility	3.52 (0.58)	3.62 (0.60)	3.51 (0.54)	3.59 (0.48)	3.67 (0.55)	3.69 (0.49)	0.79
ASA-treatment planning	3.88 (0.58)	3.86 (0.59)	3.80 (0.44)	3.86 (0.44)	3.91 (0.60)	3.95 (0.53)	0.80
ASA-practicality	3.49 (0.66)	3.74 (0.61)	3.60 (0.63)	3.60 (0.67)	3.79 (0.73)	3.93 (0.50)	0.78
MFA-benefit	4.19 (0.46)	4.18 (0.53)	4.09 (0.41)	4.18 (0.50)	4.17 (0.50)	4.16 (0.50)	0.89
MFA-harm	2.32 (0.70)	2.31 (0.75)	2.37 (0.62)	2.26 (0.64)	2.05 (0.66)	2.22 (0.74)	0.84
EBPAS total	3.03 (0.46)	3.04 (0.48)	2.98 (0.47)	3.11 (0.45)	3.05 (0.44)	3.00 (0.41)	0.79
SOF-growth	3.45 (0.70)	3.24 (0.79)	3.19 (0.74)	3.23 (0.83)	3.13 (0.65)	3.09 (0.83)	0.75
SOF-efficacy	4.01 (0.39)	3.97 (0.37)	3.98 (0.37)	3.94 (0.36)	3.95 (0.39)	3.90 (0.38)	0.52
SOF-influence	3.44 (0.61)	3.46 (0.62)	3.43 (0.65)	3.54 (0.68)	3.65 (0.68)	3.65 (0.82)	0.84
SOF-adaptability	3.80 (0.47)	3.79 (0.33)	3.85 (0.46)	3.92 (0.51)	3.82 (0.52)	3.77 (0.59)	0.54
SOF-mission	3.59 (0.50)	3.53 (0.60)	3.40 (0.65)	3.49 (0.62)	3.52 (0.57)	3.52 (0.56)	0.70
SOF-cohesion	4.18 (0.45)	3.93 (0.57)	3.86 (0.51)	3.91 (0.55)	3.83 (0.60)	3.81 (0.62)	0.81
SOF-autonomy	3.67 (0.41)	3.60 (0.45)	3.48 (0.44)	3.61 (0.47)	3.57 (0.47)	3.57 (0.47)	0.42
SOF-communication	3.35 (0.74)	3.09 (0.77)	3.00 (0.66)	3.13 (0.77)	3.06 (0.64)	2.96 (0.83)	0.80
SOF-stress	3.16 (0.80)	3.52 (0.84)	3.54 (0.80)	3.78 (0.84)	3.78 (0.66)	3.88 (0.71)	0.82
SOF-change	3.54 (0.48)	3.32 (0.47)	3.30 (0.53)	3.32 (0.67)	3.38 (0.55)	3.25 (0.54)	0.65
SOF-burnout	2.39 (0.54)	2.62 (0.61)	2.53 (0.71)	2.66 (0.63)	2.85 (0.68)	2.84 (0.76)	0.69
SOF-satisfaction	4.08 (0.52)	3.81 (0.59)	3.72 (0.61)	4.09 (0.51)	3.87 (0.55)	3.80 (0.68)	0.78
SOF-focus on outcomes	3.60 (0.49)	3.38 (0.74)	3.40 (0.65)	3.38 (0.65)	3.44 (0.49)	3.50 (0.51)	0.66
Implementation climate scale	1.85 (0.73)	1.65 (0.70)	1.58 (0.73)	1.86 (0.65)	1.77 (0.68)	1.71 (0.71)	0.78
**Structure & process**							
B&F program-level structures	2.94 (0.99)	2.88 (0.86)	2.74 (0.70)	2.66 (0.87)	3.50 (0.70)	3.09 (0.78)	0.81
B&F job-related structures	2.78 (0.92)	2.94 (0.77)	2.98 (0.71)	2.35 (0.71)	2.82 (0.61)	2.60 (0.66)	0.76
**Resources**							
SOF-program needs	3.47 (0.62)	3.38 (0.69)	3.22 (0.73)	3.52 (0.65)	3.41 (0.62)	3.27 (0.71)	0.81
SOF-training needs	3.24 (0.72)	2.98 (0.72)	2.93 (0.77)	3.09 (0.65)	2.96 (0.73)	2.79 (0.70)	0.85
SOF-offices	3.75 (0.71)	3.69 (0.64)	3.56 (0.79)	3.65 (0.67)	3.63 (0.54)	3.65 (0.52)	0.62
SOF-staffing	3.22 (0.67)	3.32 (2.28)	2.95 (0.62)	2.75 (0.52)	2.74 (0.53)	2.68 (0.54)	0.63
SOF-training	2.94 (0.70)	2.70 (0.81)	2.54 (0.81)	3.03 (0.89)	2.70 (0.75)	2.84 (0.82)	0.64
SOF-computer access	3.96 (0.45)	3.90 (0.44)	3.98 (0.45)	3.89 (0.47)	3.90 (0.41)	3.99 (0.42)	0.53
SOF-e-communications	3.80 (0.54)	3.82 (0.61)	3.80 (0.47)	3.75 (0.60)	3.71 (0.51)	3.79 (0.57)	0.27
**Policies & incentives**							
SOF-pressures for change	3.22 (0.46)	2.96 (0.63)	3.10 (0.44)	3.08 (0.46)	3.20 (0.53)	3.16 (0.41)	0.47
**Networks & linkages**							
SOF-director leadership	3.90 (0.60)	3.67 (0.82)	3.58 (0.85)	3.85 (0.84)	3.76 (0.84)	3.49 (0.93)	0.93
SOF-peer collaboration	3.75 (0.52)	3.59 (0.45)	3.50 (0.57)	3.58 (0.50)	3.53 (0.51)	3.56 (0.55)	0.48
SOF-deprivatized practice	2.93 (0.91)	2.72 (0.88)	2.77 (0.94)	2.96 (0.90)	2.91 (0.80)	3.02 (0.87)	0.74
SOF-collective responsibility	3.69 (0.54)	3.61 (0.54)	3.64 (0.63)	3.69 (0.56)	3.69 (0.59)	3.74 (0.45)	0.82
SOF-reflective dialogue	3.41 (0.61)	3.37 (0.74)	3.34 (0.77)	3.37 (0.69)	3.44 (0.64)	3.48 (0.59)	0.67
SOF-counselor socialization	3.50 (0.84)	3.41 (0.88)	3.54 (0.74)	3.55 (0.83)	3.45 (0.83)	3.40 (0.77)	0.42
**Media & change agents**							
B&F agency leadership support	3.36 (0.84)	3.37 (0.78)	3.27 (0.64)	3.06 (0.71)	3.58 (0.61)	3.32 (0.64)	0.82
Implementation leadership scale	2.85 (0.68)	2.49 (0.84)	2.63 (0.83)	2.64 (0.83)	2.55 (0.92)	2.53 (0.90)	0.85
SOF-training satisfaction	3.16 (1.01)	2.87 (1.11)	2.64 (1.05)	3.28 (1.07)	3.05 (1.08)	3.16 (1.11)	0.84
SOF-training exposure	1.52 (0.79)	1.26 (0.72)	1.39 (0.78)	1.69 (0.88)	1.53 (0.82)	1.41 (0.77)	0.69
SOF-training utilization—individual level	3.35 (0.56)	3.35 (0.54)	3.42 (0.47)	3.46 (0.79)	3.43 (0.70)	3.48 (0.59)	0.79
SOF-training utilization—program level	2.74 (0.67)	2.58 (0.84)	2.80 (0.89)	2.82 (0.75)	2.87 (0.70)	2.87 (0.81)	0.69

*ASA* Attitudes Towards Standardized Assessment, *MFA* Measurement Feedback Attitudes Scale, *SOF* Survey of Organizational Functioning, *B&F* Barriers and Facilitators Scale, *EBPAS* Evidence-Based Practice Attitudes Scale

### Quantitative analyses

We assessed group main effects and group-by-time interactions across the sustainment phase using generalized linear mixed models. Following recommendations from Singer and Willett [25], we evaluated three unconditional growth models (i.e., models with only temporal variables) to determine the trajectory that best fits the data prior to adding other fixed effects. We compared three unconditional growth models, linear, quadratic, and log-transformed, and selected the best-fitting model on the basis of the Bayesian Information Criterion (BIC). As a final step, we allowed time slopes to vary by each of the random effects and select the model with the lowest BIC. After establishing the unconditional growth model, we fit a model in which the implementation group was added as the main effect and a model in which a group-by-time interaction was added. All data analysis was conducted using R 4.0.3 in RStudio 1.4.1103 [26]. All models were fit using the glmer function in the R lme4 package. PHQ-9 completion was regressed on the mediator in a model that contained intercepts random on the patient, clinician, and clinic, which reflected multiple observations (i.e., sessions) within patients, multiple patients within clinicians, and multiple clinicians within organizations. Models assumed a binary distribution with a logistic link function. Each session represented an observation in which PHQ-9 completion was recorded.

We examined mediation effects in which contextual factors mediate the impact of the implementation group on PHQ-9 completion. Mediators were obtained at the 5-month assessment (end of active implementation), and patient outcomes were data points collected between the 5- and 15-month assessments (during sustainment monitoring). This approach allowed us to establish temporal precedence in the path of the indirect effects in which exposure to the implementation group preceded the assessment of mediators and mediator assessment preceded outcomes. To assess mediation, we tested whether Path a: implementation group predicted differences in the mediator; Path b: the mediator predicted change in PHQ-9 completion; and Path c: group predicted change in the outcome. The first two conditions enumerated above are the core relationships for establishing mediation [27]. Prior to assessing indirect effects, we assessed each of the *a* and *b* paths in individual models. All data analysis was conducted using R 4.0.3 in RStudio 1.4.1103 [26]. All *a* path models were fit with the lmer function in the R lme4 package, version 1.1.26 [28], and the degrees of freedom were estimated using Satterthwaite’s degrees of freedom method implemented using the R merTools package, version 0.5.2 [29]. In each *a* path model, the mediator was regressed on the implementation group in a model that contained an intercept random on the clinician’s clinic. Each *b* path model was fit using generalized linear mixed models with the same structure described above for PHQ-9 completion. If significant effects in the *a* and *b* paths were observed, indirect effects (i.e., the product of paths a and b) were tested using biased-corrected bootstrapped confidence intervals.

Each null hypothesis significance test is treated individually, and thus, alpha levels are adjusted, which effectively treats each test individually, as opposed to a disjunctive test in which the rejection of any of multiple null hypothesis significance tests would result in the rejection of a joint intersection null hypothesis. Alpha-level adjustments are not required for individual tests but are required for disjunctive tests [30]. Although alpha levels are not adjusted, the possibility of a type I error that can occasionally occur with a multiplicity of tests is an acceptable risk when balanced with type II errors. In accepting this risk [31], we acknowledge that the analyses are exploratory in nature.

### Qualitative methods

#### Data collection

To complement the traditional quantitative mediation analysis, we used qualitative data to surface and explore possible novel mediators, sampling for extreme variation of our target implementation outcome. The qualitative data reported here are drawn from focus group (FG) data collection at the 5-month timepoint to be consistent with the quantitative candidate mediator data. Four clinics were selected based on their variation across the implementation group (tailored/standardized) and trajectory of PHQ-9 completion during sustainment (increased/decreased). Clinics 11 (standardized) and 12 (tailored) evidenced increased PHQ-9 completion over time. Clinics 4 (standardized) and 10 (tailored) evidenced decreased PHQ-9 completion over time. Participating clinicians were predominantly female (61.1%), non-Hispanic White (61.1%) or Black (22.2%) and on average 38.6 years old (*SD* = 10.4). More than half of clinicians were licensed (55.6) and most identified their theoretical orientation as cognitive behavioral (55.6%) followed by integrative (16.7%). On average, clinicians had been working in the mental health field for 4.4 years (*SD* = 1.5).

A semi-structured FG guide was used to facilitate data collection. The guide was structured around the six domains of the Framework for Dissemination [24]. The study team saw gaps in quantitative measures for the framework’s core domains that qualitative could possibly fill more appropriately (e.g., policies, incentives). Participating clinicians were identified through purposive sampling [32] for variation in attitudes toward MBC in collaboration with the clinic director. Data collection was conducted by members of the study team trained in qualitative methods. Each FG lasted approximately 1 h; all were digitally recorded and transcribed verbatim.

#### Data analysis

Analysis occurred in an iterative and team-based process involving a directed qualitative content approach and reflexive team analysis [33, 34]. Transcripts were independently read multiple times by three qualitative analysts on the study team to achieve immersion prior to code development. Codes were derived both deductively and inductively, in order to characterize clinician experiences including both barriers and facilitators. Deductive a priori codes were based on the aforementioned core domains of the Framework for Dissemination that informed the FG guide. Inductive codes were wholly emergent from the data. Codes were independently applied by each analyst to 10% of the transcripts. Inter-coder reliability was then assessed, and following the resolution of any disagreements through discussion, the remaining transcripts were each coded by two analysts using the final coding schema [33]. Throughout the analytic process, team members met regularly to discuss emergent codes and themes and to assess the preliminary results [35]. Careful attention was given to the presence or absence of new and emerging themes throughout the analysis, and thematic saturation was achieved. Throughout the analytic process, the qualitative data software program ATLAS.ti version 7.0 was used for data organization and management.

## Results

### Quantitative mediation

The comparison of the linear, quadratic, and log-transformed unconditional growth models that were fit as a subsequent step to evaluating the implementation group on PHQ-9 completion indicated that the linear time effect with random slopes for patient and clinician was the best fitting model, see Table 5. The implementation group effects model indicated that levels of PHQ-9 completion did not differ across the sustainment phase (*z* = 0.30, *p* = 0.765). The group-by-time interaction model revealed a significant interaction across the sustainment phase (*z* =  − 2.44, *p* = 0.015). We probed the interactions with simple effects for treatment conditions at the beginning and end of the sustainment window and with simple slopes for the standardized and tailored groups. PHQ-9 completion was higher at the beginning of the sustainment window for the tailored group and was higher at the end of the sustainment window for the standardized group; however, there were no significant differences between the groups at either time point. Similarly, the tailored group exhibited a decreasing simple slope and the standardized condition exhibited an increasing slope, though neither simple slope was significant. Thus, the interaction represents a crossover interaction whereby the tailored group decreased across the sustainment window and the standardized group increased.

**Table 5:** Model results: Model 1—main effects model

Term	Estimate	SE	Statistic	*p* value
Intercept	− 1.87	0.67	− 2.78	0.005
Time (months)	− 0.05	0.49	− 0.1	0.924
Tailored	0.28	0.93	0.3	0.765
Model 2: group-by-time interaction model				
Intercept	− 2.16	0.69	− 3.13	0.002
Time (months)	1.23	0.7	1.76	0.078
Tailored	0.78	0.96	0.82	0.415
Time × tailored	− 2.32	0.95	− 2.44	0.015

Means (*SD*) and Cronbach’s alpha for each mediator are displayed in Table 4. Prior to fitting inferential models, we evaluated each of the reliability constructs using Cronbach’s *α*. We excluded scales with *α* < 0.60, which we set as the lower limit for exploratory research [36]. Excluded scales included several from the SOF.

Results for the mediation paths are displayed in Table 6. Regarding Path a, controlling for baseline levels of the outcome, the tailored group exhibited higher ratings for program-level structures on the B&F than did the standardized group at 5 months (*t*[6] = 3.84, *p* = 0.005), suggesting greater levels of facilitation of implementation at the program or clinic level. Similarly, the tailored group exhibited greater perceptions that change was possible and encouraged, and training utilization typically occurred at the program level on the SOF than did the standardized group at five months (*t*[114] = 2.29, *p* = 0.024; *t*[113] = 1.99, *p* = 0.049). Regarding Path b, only the 5-month “practicality” assessed on the ASA was a significant predictor of PHQ-9 completion across the sustainment phase of the study (*z* = 1.96 *p* < 0.050).

**Table 6:** Mediation paths using clinic-specific tailored guideline criteria

Determinant	Path A	Path B
**Norms & attitudes**		
ASA-clinical utility	0.02	0.79
ASA-treatment planning	0.07	*0.78*
ASA-practicality	− 0.01	***0.77***
MFA-benefit	− 0.01	0.73
MFA-harm	* − 0.22*	− 0.57
EBPAS total	− 0.02	0.91
SOF-growth	− 0.01	0.26
SOF-influence	0.14	− 0.26
SOF-mission	0.05	− 0.28
SOF-cohesion	0.06	− 0.35
SOF-communication	0.10	− 0.4
SOF-stress	− 0.06	0.46
SOF-change	***0.18***	− 0.73
SOF-burnout	0.09	0.06
SOF-satisfaction	0.06	− 0.14
SOF-focus on outcomes	0.17	− 0.36
Implementation Climate scale	0.11	− 0.03
**Structure & process**		
B&F program-level structures	0.70*	0.38
B&F job-related structures	0.03	0.41
**Resources**		
SOF-program needs	− 0.04	− 0.16
SOF-training needs	− 0.02	− 0.16
SOF-offices	− 0.01	* − 0.87*
SOF-staffing	− 0.33	0.09
SOF-training	− 0.04	0.08
**Networks & linkages**		
SOF-director leadership	0.15	− 0.26
SOF-deprivatized practice	0.24	− 0.21
SOF-collective responsibility	0.08	0.03
SOF-reflective dialogue	0.09	− 0.18
**Media & change agents**		
B&F agency leadership support	0.33	0.44
Implementation leadership scale	0.20	− 0.08
SOF-training satisfaction	0.09	0.27
SOF-training exposure	0.21	0.48
SOF-training utilization—individual level	− 0.01	*0.71*
SOF-training utilization—program level	***0.23***	0.17

*Note.* Italics < .10; bold < *.05*; * < *.*01

*ASA* Attitudes Towards Standardized Assessment, *MFA* Measurement Feedback Attitudes Scale, *SOF* Survey of Organizational Functioning, *B&*F Barriers and Facilitators Scale, *EBPAS* Evidence-Based Practice Attitudes Scale

### Qualitative results

Thematic analysis of the 5-month FG with the four exemplar clinics illuminates additional clinic-level characteristics that may help to explain the quantitative results. Three themes emerged from qualitative analysis of these clinics that suggest the central importance to implementation outcomes (i) of compelling communication from leadership, (ii) supportive supervision, and (iii) clinical consultation opportunities. A fourth potential theme, the presence of strong support staff, also emerged from the analysis, although it was only explicitly discussed at one clinic. Each of these themes is discussed further below with quotes provided in Table 7.

**Table 7:** Exemplar quotes for qualitative results

Site (implementation group/PHQ-9 trajectory)	Quote
Compelling leadership communication	
11 (standard/increasing)	Just communicating [was important for implementation experience at this clinic]. Just when [leadership] rolls something out, them communicating with us, giving us education, so we know how to use and how we're supposed to use it and know what the purpose is.
12 (tailored/increasing)	Systemic operational environment I think is very important. It has a big effect on how successful this is because I think logistical things are a big barrier or help. I think my viewpoint as a team lead is, I see and have hope that the organization is trying to move to not just pure productivity numbers, but also some measurement based care… they do encourage the PHQ-9. [The message is that] it's important, because it's good for the patient.
4 (standard/decreasing)	If they would just explain to us how this could be useful for us and useful for our consumers I think we would have been more receptive to it, but a lot of times that's not what happens. They tell us what it is that they want us to do and we're standing around like a three year old. "But why?" We never get the answer.
10 (tailored/decreasing)	Clinician 1: It’s always, “Here’s something new, you gotta do this now.” But it’s not, “this is going to replace that.” Clinician 2: And it’s not why! There’s no why Clinician 1: Yeah, there’s hardly ever a why and it’s… Clinician 3: In addition to Clinician 4: And you’ll get penalized if you don’t do it!
Supportive supervision	
11 (standard/increasing)	When your clinic manager -and I’m thinking also maybe like the regional manager- if they support what you’re doing and they’re helping to facilitate what’s being asked, I see that as a plus. And it’s more likely then that, whether it’s measurement-based care of some other, it’s gonna be implemented and implemented well. Without that support, if you’re being asked to do something, and you feel like you don’t have that support, that can be a little discouraging.
12 (tailored/increasing)	Clinician 1: Our direct supervisor really helps facilitate PHQ-9. I know she's really good about reminding us to do them, so she's been a huge help. She even brought us all copies of the paper forms so we'd have them to use, so I think she's probably the biggest support we have for it. Clinician 2: Which encourages us to use it. Frankly, if anyone above her mentioned it, it wouldn't have any effect on my behavior. I don't know any of them personally above her…. I've met [the Vice President], but apart from that, I don't know who runs this place, and I don't care.
4 (standard/decreasing)	Clinician 1: We had confused supervision a little bit. Especially early on, I think we were being told different things Clinician 2: My clinical supervisor didn't know what it was. I had to explain it to her and showed it to her. She was like, "oh, okay." She travels between offices, so … She doesn't see patients, I don't think, so I don't know. Facilitator: Okay, so your supervision didn't really include that element. Anyone else have supervision where it helped, hurt, wasn't present? Clinician 3: Yeah, I have supervision. Of course I vented about it, about us having to implement this and then it went from doing it voluntarily now it's mandated to continue to do it. She just like, "Well I really don't have anybody to do this." Is what she said.
10 (tailored/decreasing)	[My clinical supervisor], she has staffing and she has so much administrative stuff she has to get done because her higher ups are telling her to get it done. We [clinicians] come in as clinical people thinking we don't want to do that, we want to talk about our patients [in supervision], we want to understand how to help them better. You can't do both when you have such a large group. So, does she get her part done, the admin stuff? Or do we get our part done, to get support? Neither get done satisfactorily. In my opinion
Clinical consultation opportunities	
11 (standard/increasing)	With the organization, with Centerstone, to have that support, that goes a long way, and again, knowing that your colleagues are kinda doing the same thing. It's been helpful, you know, the tri-weekly meetings that we've had over the telephone, I've enjoyed those. That's supportive because you're getting feedback and things like that about a different concept.
12 (tailored/increasing)	I feel like, especially in the children's department, right now we have five clinicians total, and then a lot of family support specialists, and so my group is much smaller, more cohesive, and I think that there's a lot more of the trust. We support each other and talk. I don't think anybody in my department would have a feeling of isolation, and so that's good for the children [patients].
4 (standard/decreasing)	Clinician 1: From a clinic perspective I don't know if there is an actual norm. We're sort of left to our own implementation devices. Facilitator: What do you see happening? Are you seeing your colleagues using measurement based care, or not so much? Clinician 1: I don't know if I'd had the opportunity to see yay or nay on that. Facilitator: Okay. You don't have a window into each other's work to know. Clinician 1: We ain't got time for that. Clinician 2: We wave at each other as we pass in the hall.
10 (tailored/decreasing)	Facilitator: Do you feel like you have chances to discuss new things that you're using? Clinician 1: With our staff? With each other? Facilitator: Yeah, with each other, to support each other? Clinician 1: I don't. We kind of, like, live in our office Clinician 2: We work in different buildings! So the only reason I have even gotten to talk to [another clinician in the focus group] is the fact that we're doing video conferencing once every three weeks for this [study] meeting. Otherwise, you and I would never talk Clinician 3: I come to your staffings, that's the only reason I see you Clinician 1: I never talk to [another clinician]
Strong support staff	
4 (standard/decreasing)	Going back to implementation measurement based care, in this particular clinic, another big barrier is, has been honestly, our support staff. We've had a lot of turnover in the past year. I might not be supposed to know this, but I go up to [different clinic] every Friday and so I get to see how they implement measurement-based care compared to us. I'm seeing their support staff giving out the surveys every time a patient checks in. I'm going, "oooh, that looks helpful." I don't know if our current front desk could handle something else on top of them right now. They're barely checking our patients in as it is

#### Compelling leadership communication

One of the most salient themes emergent in the analysis was the importance of effective and compelling leadership communication about the messaging around MBC. Clinicians viewed favorably, and were motivated to action by, leadership communication that provided clear incentives and/or rationale for the adoption of MBC at their clinics and thus contextualized its implementation. In contrast, leadership communication that was experienced as a top-down directive, delivered without appropriate explanation or contextualization, was viewed unfavorably and resulted in resentment and cynicism. In the four exemplar clinics analyzed, clinicians at both Clinic 11 (standard/increased PHQ-9 Completion) and Clinic 12 (tailored/increased PHQ-9 Completion) reported compelling communication from leadership. Conversely, clinicians at Clinic 4 (standard/decreased PHQ-9 Completion) and Clinic 10 (tailored/decreased PHQ-9 Completion) reported messaging from clinic leadership that failed to provide a clear or compelling rationale for the implementation of MBC.

#### Supportive supervision

Another emergent theme in the qualitative analysis was the critical role of the supervisor in implementation uptake. Effective, supportive supervision, as evidenced by supervisors’ willingness to devote time to clinical and logistical details relevant to clinicians’ implementation of MBC and their provision of encouragement and support, was described by clinicians at both Clinic 11 and Clinic 12. In contrast, clinicians at Clinics 4 and 10 described supervision that was lacking in these areas.

#### Clinical consultation opportunities

A third emergent theme in the focus group analysis focused on differential opportunities across clinics for clinicians to connect for consultation with colleagues. In clinics that provided such opportunities, including Clinics 11 and 12, clinicians were able to compare notes about MBC, provide and receive support and tips, and enjoy a sense of professional community. However, in clinics where such opportunities were not present, including Clinics 4 and 10, clinicians reported experiencing a sense of isolation that impeded the chance for professional support.

#### Strong support staff

A fourth potential mediating characteristic, the importance of strong support staff, also emerged in the analysis. Because it was explicitly discussed only by one clinic, it falls short of a theme, but we include it here because clinicians at one clinic, Clinic 4, reported the impact of its absence and openly compared it with another clinic whose staff they perceived as more helpful in supporting the implementation of MBC.

## Discussion

In the present study, no significant mediators emerged from our quantitative analyses, but several partial pathways point toward critical areas for future research and might inform advancements in implementation theory. Our exploratory qualitative analyses tell a story of the importance of support from colleagues, supervisors, and leadership when implementing clinical innovations in practice.

### Quantitative results

This study is one of few longitudinal implementation trials with rich quantitative data to engage in rigorous mediation analyses. The experimental design of this study also fulfilled several key requirements for identifying mediators including variable selection guided by implementation theory, longitudinal design in which changes in predictors preceded changes in outcomes across time, random assignment to a group, and experimental manipulation of groups [13]. Although no quantitative mediators emerged, three scales demonstrated significant improvement from baseline to 5 months in the tailored group. These were program-level training utilization and organizational climate for change from the SOF [37] and program-level structures from the B&F [38]. These constructs assess clinician perception of the extent to which new interventions are supported and used at the organizational level. More specifically, the program-level training utilization scale assesses clinician perception of the frequency at which new interventions are integrated into the organization following training (e.g., “How often do new ideas learned from workshops get discussed or presented at your staff meetings?”). The organizational climate change scale assesses organizational expectations and encouragement of change (e.g., “You are encouraged here to try new and different techniques”), and the program-level structure scale assesses organizational infrastructure to support MBC (e.g., “We use fidelity reports to improve our MBC practice”).

A deeper look at how the tailored group unfolded over time provides possible explanations for why these scales were *not* associated with PHQ-9 completion from 5 to 15 months. During the 5-month active implementation period, clinics in the tailored group held approximately triweekly implementation team meetings. Implementation teams were tasked with selecting and executing strategies to facilitate MBC implementation. As reported in a previous study of these implementation teams’ strategy use, quality management and communication were among the most common categories of strategy use [39]. Quality management took the form of review and discussion of reports of MBC use at the clinic (i.e., “fidelity reports”) and communication strategies often took the form of encouragement to use the PHQ-9 in clinic team meetings, the break room, and via email. When strategies used by the implementation team are compared with individual items from the above scales, it seems possible that clinicians at 5 months were reflecting on the efforts of their clinic implementation team. However, following the 5-month active implementation phase, the study team withdrew active facilitation support. Although some clinics opted to continue meeting, it was typically with reduced frequency and intensity. It is possible that across the next 10 months, these strategies were not consistently used undermining lasting changes in MBC use. Community mental health clinicians are faced with competing and constantly changing demands. For example, as is common in many community mental health organizations, clinicians expressed concern about achieving productivity requirements for billing in focus groups and informally to study staff during training visits and meetings [40]. Continuing to devote unbillable time to implementation team meetings and to-dos generated during meetings might have felt unfeasible. If these program-level strategies were contingent upon the active support of the study team, then its discontinuation may well have undermined MBC sustainment [41].

This raises the question: does tailoring change the context or does tailoring align strategies to be responsive to context and accommodate the barriers to implementation? As an analogy, if a person has the intention to go for a walk but has a broken leg, crutches can be used as a strategy to support the person to go for a walk. However, when this aid is removed, the person can no longer go for a walk. The context (broken leg) remains unchanged, but strategy (crutches) supports movement toward the goal (going for a walk). In parallel, once active study team support was removed, clinics that previously struggled with unclear communication from leadership, administratively focused supervision, and competing demands were faced with these barriers again without the “crutch” of the implementation team. Successful repair of a broken leg that enables a person to walk freely requires setting and bracing the leg as well as taking weight off the leg for a sufficient period of time to heal. In parallel, it could be that strategies to permanently alter (setting and bracing the leg) and/or lengthier involvement by the study team (using the crutches for an extended period of time) are needed to sustain the use of measurement-based care. In the tailored group, the clinic team members were empowered by the study team to direct implementation strategies given their site-specific expertise. However, teams resorted to the use of a limited number of all possible implementation strategies that may not have supported sustainment optimally [39]. Although clinic implementation team members were experts in their own work context, understandably, they were not experts in the science (or practice) of implementation and thus not knowledgeable about more intensive strategies geared toward leadership and managerial change [42].

There are specific packages of implementation strategies such as the Leadership and Organizational Change for Implementation (LOCI) intervention that have been shown to result in alterations in clinician perceptions of leadership style and leadership support for implementation efforts involving repeated training sessions, coaching calls, feedback reports about leadership, and individualized development plans to improve leadership [43, 44]. Although clinic leadership and informal leaders (i.e., opinion leaders) were included on implementation teams, the present study did not make explicit attempts to alter individuals’ approaches to leadership or supervision. And, in fact, candidate mediators reflecting aspects of leadership (i.e., Director Leadership subscale from the SOF, ILS, and Agency Leadership Support subscale from the BFS) did not change as a result of tailoring during the 5-month active implementation period.

In addition to the three scales that demonstrated significant improvement from baseline to 5 months in the tailored group, the practicality subscale of the ASA measure at 5 months was associated with PHQ-9 completion at 15 months. The practicality subscale captures perceptions of the ease of using measures like the PHQ-9 (e.g., “standardized progress measures can efficiently gather information”), meaning that clinicians who found the PHQ-9 easy to use were more likely to sustain administration over time. As a reminder, the standardized group was not an inert control, rather it represented certain implementation “best practices” which included support for the use of MBC via triweekly consultation meetings. Consultation has been shown to be a promising strategy for supporting clinician skill acquisition and implementation of new practices [45]. It is therefore possible that clinicians across groups who used MBC during the 5-month active implementation period gained direct exposure to the ease of use and continued to incorporate it in their practice through the sustainment period. It is somewhat surprising that the experimental group (i.e., tailored or standardized group) did not alter perceptions of practicality across the 5-month active implementation period given the concerted effort by several implementation teams in the tailored group to incorporate PHQ-9 administration into the front-office workflow. However, when office professionals administered the PHQ-9 at patient check-in, clinicians may have not been directly exposed to the speed and efficiency of measure completion.

### Qualitative results

Given the variability in the trajectories of PHQ-9 completion during the sustainment phase that could not be explained by the study group, we undertook exploratory qualitative analyses of the focus groups conducted at the end of the 5-month active implementation period to characterize two exemplar clinics that demonstrated improvement in PHQ-9 completion versus two clinics that deteriorated regardless of the study group (see Table 8 for characteristics of exemplar clinics). A comparison of improving and deteriorating clinics revealed the role of leadership, supportive supervision, and clinical consultation with peers in the implementation of MBC. Specifically, clinicians at clinics that experienced increases in PHQ-9 completion across both the standard and tailored groups described clear communication from leadership regarding expectations and rationale for the implementation of MBC, incorporation of MBC discussion during clinical supervision, and opportunities to consult about MBC or receive general support from colleagues as facilitative. Conversely, clinicians at clinics that experienced decreases in PHQ-9 completion described unclear top-down (or no) communication about MBC from leadership, confusion about MBC from supervisors, and isolation from colleagues. These findings map onto extant implementation research that demonstrates the importance of strong leadership and the role of supervisors as leaders in supporting implementation efforts and skill acquisition [46–48]. The role of peer support is also reflected in the literature regarding the influence of social norms on behavior [49].

**Table 8:** Qualitative themes and characteristics of clinics

*Characteristic*	Description	Site 12	Site 11	Site 10	Site 4
State		IN	TN	IN	TN
Rural/urban status		Urban	Urban	Rural	Urban
Clinic size	Based on # of therapists employed at time of cohort assignment; Small < 15; medium = 16–20; large = > 20	Large	Medium	Medium	Small
Trajectory of PHQ9 use	Increasing or decreasing use of PHQ9 across 15 mo	Increasing	Increasing	Decreasing	Decreasing
Study condition	Standard vs. tailored	Tailored	Standard	Tailored	Standard
Effective leadership	Clear incentive/rationale for MBC use provided by leadership (i.e., not just top-down directive)	Yes	Yes	No	No
Effective supervision	Time in supervision devoted to clinical and logistical details, and kudos/encouragement provided	Yes	Yes	No	No
Clinical consultation with colleagues	Opportunities to provide and receive support and enjoy a sense of professional community	Yes	Yes	No	No

Although attempts were made to capture some of these domains, particularly leadership, via the quantitative data, baseline levels of these measures were controlled for when quantitatively assessing the association between the experimental group and candidate mediators, meaning it was not possible to determine if pre-study strong/weak leadership and supervision played a role in PHQ-9 completion. In addition, measures that assessed leadership or clinician perceptions of the organization (i.e., ILS and certain subscales of the SOF) did not orient the survey-taker to the level of analysis to which they should respond. For this reason, it is possible that some clinicians responded to survey questions with their direct supervisor in mind while others may have responded with upper-level organizational leadership in mind. This is especially possible given that some clinics housed regional leadership making them more visible to clinicians compared with clinics who were more disconnected from upper-level leadership. Future studies should, therefore, carefully define the level of analysis in the survey directions and, when appropriate, in the body of the survey [50].

### Limitations and implications for implementation strategy mediation analyses

There were several limitations to the current study that surface implications for future implementation strategy mediation analyses. First, there are a host of measurement issues, such as the one described above in which measures that assessed leadership or clinician perceptions of the organization did not orient the respondent to the level of analysis to which they should respond. Thinking carefully about the theory of change and the target social unit for an implementation strategy will be important for devising measure instructions because no single study could assess perceptions of all stakeholder leader-like roles. Moreover, prior research has demonstrated that manipulation of the EBPAS language, for example, to refer to general interventions versus specific evidence-based treatments changes clinician response suggesting the importance of assessing intervention-specific attitudes rather than general attitudes [51]. Although we deployed surveys multiple times, it is possible that waiting some period of time post-active implementation support is crucial when measuring candidate mediators; guidance is needed. Moreover, even though our survey battery of relevant contextual factors was quite extensive, measures related to patients, their perspective, and the clinical encounter itself (e.g., length of time, purpose) [52] were altogether missed.

Although qualitative inquiry presents a way in which to surface novel contextual factors where quantitative measures do not exist, the focus group interviewing guide used in this study queried the same domains outlined in the Framework for Dissemination [24] and, as such, may have missed other relevant contextual factors not included in this framework. We did offer clinicians an opportunity at the end of the focus group to share about areas not queried in the main portion, but relatively little new information emerged at that time. In addition, although attempts were made to include clinicians who had varying use and opinions about MBC, it is possible that capacity and willingness to attend resulted in a biased sample of respondents. Rapid ethnographic methods might offer a way to overcome some of the limitations of the traditional focus group methodology, which might be important for the study of factors influencing sustainment [53].

Finally, this approach to tailoring the implementation of measurement-based care, although informed by a framework, was largely atheoretical. The field is at a point of readiness for centering theory to inform practical implementation [54, 55]. In the case of tailoring implementation, causal theory can be leveraged in at least two ways. One, determinants rarely act in isolation yet commonly used approaches to prioritizing barriers for tailoring strategies invite stakeholders to rate each individual factor for its importance and/or feasibility, among other parameters [1]. Bringing theory to bear on this step, to generate a theory of the problem, could result in a more focused set of determinants. This could be done by leveraging the notably sparse implementation theory or by theorizing the temporal interrelationships among local contextual factors and implementation outcomes [56, 57]. Two, strategies could be matched or tailored to determinants by articulating their mechanisms of action and aligning those with target barriers. This could be done by diagramming causal pathways of strategies that center determinants, although more empirical work, is needed to bring confidence to this endeavor [58].

## Conclusions

This study explored candidate mediators of MBC sustainment using a longitudinal design that enabled rigorous quantitative analyses mixed with qualitative methods for additional insight. No mediators were established using quantitative methods perhaps due to poor or insufficient measurement, or failure of the implementation strategies to fully resolve determinants and institutionalize MBC. Several partial pathways were identified pointing to fruitful areas for future research. Specifically, quantitative analyses revealed the association between implementation group and program-level training utilization, organizational climate for change, and program-level structures as well as the association between clinician perception of MBC practicality and sustainment of PHQ-9 completion over time. Future research should consider experimental manipulations to further explore the role of practicality and user experience in sustained evidence-based practice use. Future research may also be needed to disentangle strategies that accommodate barriers to implementation versus permanently altering or removing barriers. Qualitative results emphasized the importance of leadership, supervision, and peer support for implementation which aligns with past research. Future research is needed to clarify pragmatic operationalizations of strategies to alter or increase the potency of these determinants particularly in the context of sustainment.

## Data Availability

A limited quantitative dataset analyzed in the current study may be available upon reasonable request made to the corresponding author.

## Abbreviations

FG: Focus group
PHQ-9: Patient Health Questionnaire
MBC: Measurement-based care
SOF: Survey of Organizational Functioning
EBPAS: Evidence-Based Practice Attitudes Scale
ASA: Attitudes Towards Standardized Assessment Scales-Monitoring and Feedback
MFA: Monitoring and Feedback Attitudes Scale
ICS: Implementation Climate Scale
ILS: Implementation Leadership Scale

## Footnotes

1 Context includes key features of the environment in which the work is immersed and which are interpreted as meaningful to the success, failure, and unexpected consequences of the intervention(s), as well as the relationship of these to stakeholders. These factors can surface as barriers or facilitators [2]

2 Factors that obstruct or enable changes in targeted professional behaviors or healthcare delivery processes [3].

3 A mediator is an intervening variable that may account (statistically) for the relationship between the independent and dependent variable. In this case, contextual factors that surface as barriers may emerge as mediators of the tailored implementation and its outcomes [13]
